# Cleavage of ST6Gal I by Radiation-Induced BACE1 Inhibits Golgi-Anchored ST6Gal I-Mediated Sialylation of Integrin β1 and Migration in Colon Cancer Cells

**DOI:** 10.1186/1748-717X-7-47

**Published:** 2012-03-27

**Authors:** Minyoung Lee, Jung-Jin Park, Young-Gyu Ko, Yun-Sil Lee

**Affiliations:** 1 Division of Radiation Effects, Korea Institute of Radiological and Medical Sciences, Seoul 139-706, South Korea; 2 College of Life Sciences and Biotechnology, Korea University, 1, 5-ka, Anamdong, Sungbuk-gu, Seoul 136-701, South Korea; 3 College of Pharmacy & Division of Life & Pharmaceutical Sciences, Ewha Womans University, 11-1 Daehyun-Dong, Seodaemun-Gu, Seoul 120-750, South Korea

**Keywords:** BACE1, Migration, Radiation, ST6Gal I

## Abstract

**Background:**

Previously, we found that β-galactoside α2,6-sialyltransferase (ST6Gal I), an enzyme that adds sialic acids to N-linked oligosaccharides of glycoproteins and is frequently overexpressed in cancer cells, is up-regulated by ionizing radiation (IR) and cleaved to a form possessing catalytic activity comparable to that of the Golgi-localized enzyme. Moreover, this soluble form is secreted into the culture media. Induction of ST6Gal I significantly increased the migration of colon cancer cells via sialylation of integrin β1. Here, we further investigated the mechanisms underlying ST6Gal I cleavage, solubilization and release from cells, and addressed its functions, focusing primarily on cancer cell migration.

**Methods:**

We performed immunoblotting and lectin affinity assay to analyze the expression of ST6 Gal I and level of sialylated integrin β1. After ionizing radiation, migration of cells was measured by in vitro migration assay. α2, 6 sialylation level of cell surface was analyzed by flow cytometry. Cell culture media were concentrated and then analyzed for soluble ST6Gal I levels using an α2, 6 sialyltransferase sandwich ELISA.

**Result:**

We found that ST6Gal I was cleaved by BACE1 (β-site amyloid precursor protein-cleaving enzyme), which was specifically overexpressed in response to IR. The soluble form of ST6Gal I, which also has sialyltransferase enzymatic activity, was cleaved from the Golgi membrane and then released into the culture media. Both non-cleaved and cleaved forms of ST6Gal I significantly increased colon cancer cell migration in a sialylation-dependent manner. The pro-migratory effect of the non-cleaved form of ST6Gal I was dependent on integrin β1 sialylation, whereas that of the cleaved form of ST6Gal I was not, suggesting that other intracellular sialylated molecules apart from cell surface molecules such as integrin β1 might be involved in mediating the pro-migratory effects of the soluble form of ST6Gal I. Moreover, production of soluble form ST6Gal I by BACE 1 inhibited integrin β1 sialylation and migration by Golgi-anchored form of ST6Gal I.

**Conclusions:**

Our results suggest that soluble ST6Gal I, possibly in cooperation with the Golgi-bound form, may participate in cancer progression and metastasis prior to being secreted from cancer cells.

## Background

ST6Gal I (β galactoside α2,6 sialyltransferase, CMP-NeuAc: Galβ (1,4) GlcNAc: α2,6 sialyltransferase) is an important glycosyltransferase that adds a sialic acid residue to the terminal position on N-linked oligosaccharides [1,2]. It is localized in the Golgi apparatus in a membrane-anchored form and is cleaved into a secretary protein by cathepsin-like proteases [3]. Recent studies and clinical reports have emphasized the importance of ST6Gal I in cancer progression and metastasis. ST6Gal I is up-regulated in colon adenocarcinoma and its expression is positively associated with tumor cell migration and invasion [4-6]. Specifically, patients with metastasizing tumors have high levels of ST6Gal I in their serum, and serum levels of ST6Gal I are correlated with the progression of colorectal carcinomas and cancer metastasis [7-13]. However, a possible biological role of ST6Gal I in the plasma has not been reported.

Metastasis represents an obligatory step in cancer progression. A variety of molecules contribute to cancer progression and metastasis [14], and many of the factors that function in tumor metastasis are glycoproteins [15-17]. It has been previously demonstrated that integrin β1 is a major substrate of ST6Gal I [4,18]. In colon epithelial cells, oncogenic Ras has been shown to up-regulate ST6Gal I expression, leading to increased α-2,6 sialylation of β1 integrin [19]. Hypersialylation of integrin β1 augments colon cancer metastasis by altering cellular preference for a certain extracellular matrix milieu as well as by stimulating cell migration. Integrins also regulate cellular functions, including survival, proliferation and cell spreading, through the function of signaling molecules co-localized to the focal adhesion complex [20,21]. We have previously demonstrated that exposure to ionizing radiation (IR) increases the expression of ST6Gal I as well as the level of sialylated glycoprotein. Sialylation of integrin β1 by exposure of cells to IR increases the adhesion and migration of colon cancer cells through integrin β1-mediated cellular signaling. Therefore, integrin β1 sialylation and the subsequent activation of p130CAS, paxillin, and AKT signaling may be one of the mechanisms involved in IR-mediated-radioresistance and cancer metastasis [22-26].

β-site amyloid precursor protein-cleaving enzyme (BACE) is a membrane-bound aspartic protease that cleaves the amyloid precursor protein (APP) in the pathogenesis of Alzheimer's disease [27,28]. Importantly, BACE has been identified as a protease responsible for the cleavage and secretion of Golgi-resident ST6Gal I [29]. The mechanisms underlying cleavage are complicated, and have not been well characterized. Soluble forms of glycosyltransferases exist in the plasma of patients with certain diseases, and can sometimes be used as biomarkers for these diseases [30-33].

In the present study, we examined IR-induced cleavage and solubilization of ST6Gal I, which is released into the cell culture media of colon cancer cell lines, and sought to identify the protease involved in cleaving ST6Gal I after exposure to IR. We found that BACE1 could be the secretase responsible for IR-induced cleavage of ST6Gal I, and showed that BACE1 mediated cleavage of ST6Gal I decreased ST6Gal I -mediated cancer cell migration. Additionally, the soluble form of ST6Gal I possesses sialyltransferase enzymatic activity, but unlike the Golgi-associated form, did not sialylate integrin β1 to a significant degree, suggesting that soluble ST6Gal I, possibly in cooperation with the Golgi-bound form, may participate in cancer progression and metastasis before being secreted from cancer cells, independent of intergrin β1-sialylation.

## Materials and methods

### Cell culture

SW480 and SW48 human colorectal carcinoma cells were grown in RPMI media supplemented with heat-inactivated 10% fetal bovine serum and antibiotics. CT26 mouse colorectal carcinoma cells were cultured in Dulbecco's modified Eagle's medium (DMEM) supplemented with heat-inactivated 10% fetal bovine serum and antibiotics.

### Plasmids and transfection

For transient transfection experiments, expression constructs of C-terminally Flag-tagged wild-type (WT; p3 × Flag-ST6Gal I) ST6Gal I and N-terminally deleted (HA-tagged) ST6Gal I (ST6Gal I ΔN) were created as described previously [23]. An N-terminally Flag-tagged double-mutant, ST6Gal I (L37A/K40A) was created by site-directed mutagenesis [34]. Predesigned small interfering RNA (siRNA) for ST6Gal I was purchased from Dharmacon (Lafayette, CO, USA). BACE-1 was targeted with the siRNA duplex 5'-AGA UCC UGU CCA UUG AU CUC CAC CC-3' and 5'-GGG UGG AGA UCA AUG GAC AGG AUC U-3' [35]. A BACE-1 expression plasmid (pcDNA3.1/BACE-1) was generously provided by Dr. Sul-Hee Chung (Inje University, Pusan, Korea) [36]. Cells were transfected with plasmids using LipofectAMINE 2000 (Invitrogen, Carlsbad, CA, USA) as described by the manufacturer.

### Irradiation

Cells were exposed to γ-irradiation using a ^137^Cs γ-ray source (Atomic Energy of Canada, Mississauga, ON, Canada) at a dose rate of 3.81 Gy/min.

### Immunoblot, lectin affinity assay, and immunoprecipitation

Protein levels were detected using the following commercial antibodies: anti-integrin β1 (BD Biosciences, Franklin Lakes, NJ, USA); anti-phospho-p130CAS, anti-phospho-Src, anti-HA, and anti-Myc (Cell Signaling Technology, Danvers, MA, USA); anti-Flag (Sigma); and anti-ST6Gal I and anti-BACE-1 (IBL, Japan). For detection of sialylated proteins, cell lysates were incubated with biotinylated SNA (*Sambucus Nigra lectin*; Vector Laboratories, Burlingame, CA, USA), and protein-lectin complexes were precipitated with avidin-coated protein A-agarose (Sigma).

### Flow cytometry

Cells were detached with trypsin/EDTA at the indicated times and stained with fluorescein isothiocyanate (FITC)-conjugated SNA (FITC-SNA) or *Maackia amurensis *(FITC-MAA) lectin (Vector Laboratories, Burlingame, CA, USA) for detection of α2,6 and α2,3 sialylation, respectively. After staining, fluorescence intensity was analyzed by fluorescence-activated cell sorting (FACS).

### Reverse transcription-polymerase chain reaction (RT-PCR)

Total RNA was isolated with TRI reagents (Molecular Research Center), and reverse transcription was done using Omniscript transcriptase (Qiagen) under the following thermocycling conditions: 30 cycles of 95°C for 5 min (denaturing), 60°C for 30 s (annealing), and 72°C for 30 seconds (extension). The primer sequences used were as follows: human BACE1, 5'-GGT GGA GAT CAA TGG ACA GG-3' (sense) and 5'-CGT GGA TGA CTG TGA GAT GG-3' (antisense); mouse Bace1, 5'-GCA GAC CCA CAT TCC GAA CA-3' (sense) and 5'-GCC ACT GTC CAC GAT GCT CTT-3' (anti-sense); and GAPDH, 5'-CAT GGA GAA GGC TGG GGC TCA TTT-3' (sense) and 5'-CGC CAG TAG AGG CAG GGA TGA TGT-3' (antisense).

### *In vitro *migration assay

Cell migration assays were performed using a Boyden chamber as previously described [24]. Cells were plated on the upper side of a collagen treated, polycarbonate membrane separating two chambers of 6.5-mm Transwell culture plates (Costar, Corning, NY, USA). After 24 hours, cells on the upper face of the membrane were scraped using a cotton swab and cells that had migrated to the lower face of the membrane were stained with DiffQuick (Baxter Scientific, Deerfield, IL, USA) Wright-Giemsa Solution.

### Soluble ST6Gal I ELISA

Cell culture media were concentrated using Centricon centrifugal filter devices (Millipore, Billerica, MA, USA) and then analyzed for soluble ST6Gal I levels using an α2,6 sialyltransferase sandwich enzyme-linked immunosorbent assay (ELISA) kit (IBL, Japan), according to the manufacturer's protocol.

### Statistical analysis

Data are expressed as means ± standard deviations (SDs). Statistical significance was determined using Student's *t*-test for comparisons between two means. The null hypothesis was rejected in cases where *p-*values were < 0.05.

## Results

### Inhibition of ST6Gal I cleavage by knockdown of BACE1 increases sialylation of cell surface protein

**Figure 1:**
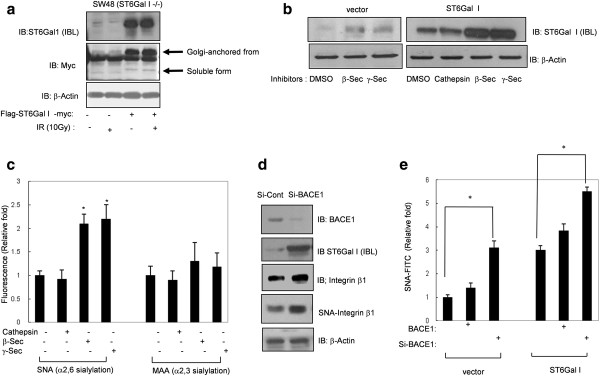
**Inhibition of BACE1 stabilizes ST6Gal I and increases cell surface sialylation**. A. SW48 (ST6Gal I-negative) colon cancer cells were transiently transfected with an N-terminally Flag-tagged ST6Gal I expression plasmid, and 24 hours later, cells were exposed to IR (10 Gy). Cell lysates were assessed for expression of ST6Gal I by immunoblotting with an ST6Gal I antibody. B. SW480 vector-control cells and cells stably expressing ST6Gal I were treated with inhibitors of β-secretase, γ-secretase, or cathepsin B&L at a concentration of 20 μM. After incubating for 24 hours, cells were harvested and ST6Gal I levels were analyzed by immunoblotting with an anti-ST6Gal I antibody. C. Cell surface α2, 6 sialylation (SNA-FITC) and α2, 3 sialylation (MAA-FITC) were analyzed by FACS after 24 hours of treatment with 20 μM β-secretase or γ-secretase inhibitors. D. SW480 cells were transfected with siRNA against BACE1, and the levels of BACE1, ST6Gal I, and integrin β1 protein were assessed by immunoblotting. Sialylation of integrin β1 was assayed by SNA lectin affinity assay. E. SW480 vector-control cells and cells stably expressing ST6Gal I were treated with Si-BACE1 or transfected with a BACE1 expression plasmid. Then, α2, 6 sialylation (SNA-FITC) of the cell surface was analyzed by FACS. Data are presented as means ± SDs of three replicates (*p < 0.05 vs. the corresponding control).

In our previous study, we found that IR exposure induces ST6Gal I cleavage and produces a soluble form of ST6Gal I in SW480 colon cancer cells [23]. To support the result of our prior work, we next examined the expression of soluble ST6 Gal I in SW48 cells (ST6 Gal I^-/-^) by transiently transfecting these cells with expression vectors for Golgi-anchored (N-terminally Flag-tagged and C-terminally Myc-tagged) of ST6Gal I and exposing them to IR. As shown in Figure 1A, both the Golgi-anchored and soluble forms of ST6Gal I were expressed in SW48 colon cancer cells (Figure 1A). Because it had previously been demonstrated that sialyltransferases are cleaved by cathepsin-like protease or BACE [3,29,34,37], we sought to identify the target protease(s) responsible for cleavage of ST6Gal I using various protease inhibitors in SW480 vector-control and ST6Gal I-overexpressing cells. Inhibitors of β-secretase and γ-secretase increased the protein levels of the uncleaved, proform of ST6Gal I, whereas cathepsin B and L (CTS) inhibitors did not (Figure 1B). In addition, α2, 6 sialylation level at the cell surface, detected by FITC-conjugated SNA, was also increased by treatment with β-secretase or γ-secretase, but not CTS, inhibitors. In contrast, α2, 3 sialylation at the cell surface, detected by FITC-MAA, was not altered by these inhibitors (Figure 1C), suggesting that β-secretase or γ-secretase might be responsible for the cleavage of ST6Gal I in SW480 colon cancer cells. Next, because BACE1 is reported to be the protease responsible for the cleavage of ST6Gal 1 [34,35], we tested the effects of siRNA-mediated BACE1 knockdown. We observed a concomitant increase in the level of non-cleaved ST6Gal I protein and sialylation of integrin β1, a substrate of ST6Gal I (Figure 1D). Moreover, knockdown of BACE1 expression augmented the sialylation on the surface of these cells. Interestingly, BACE1 overexpression did not specifically inhibit cell surface sialylation (and may have slightly increased it) compared with control levels (Figure 1E), suggesting that BACE1-mediated cleavage of ST6Gal I only slightly shows the sialylation potential of ST6Gal I. Taken together, these results suggest that BACE1 may be responsible for cleavage of ST6Gal I.

### The non-cleavable, double-mutant ST6Gal I (L37A/K40A) increases α2, 6 sialylation at the cell surface

**Figure 2:**
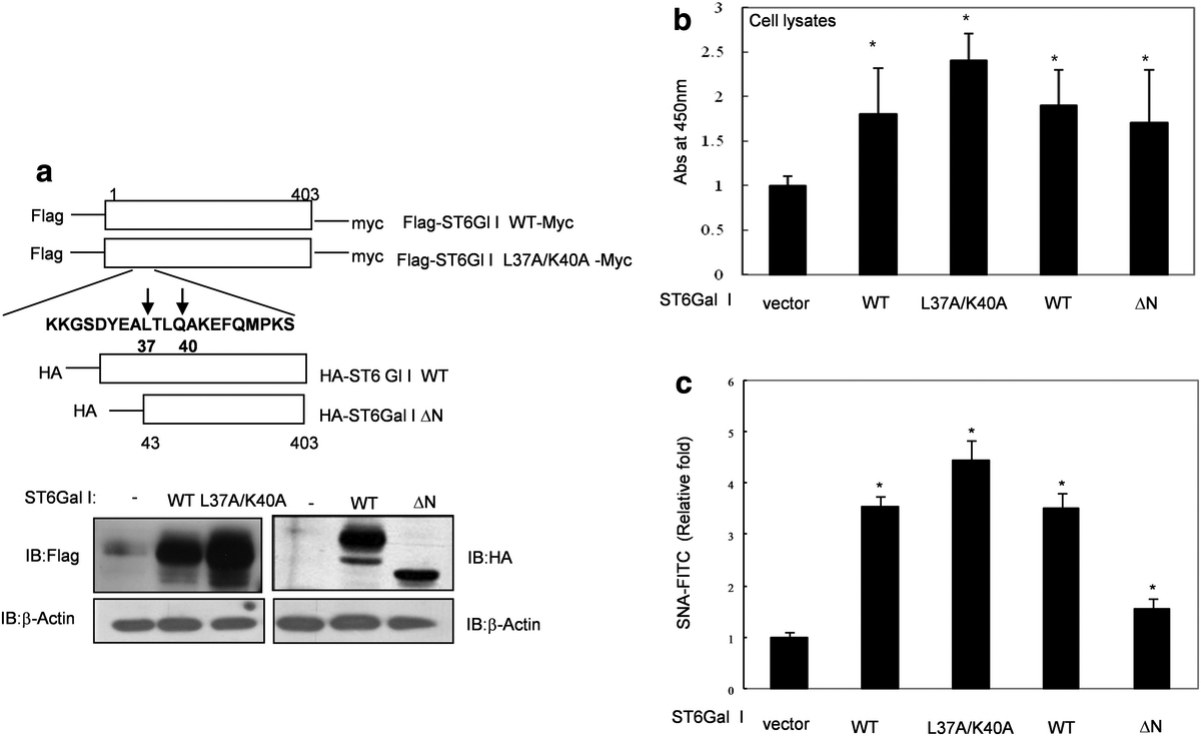
**Construction of ST6Gal I expression plasmids and their effect on α2, 6 sialylation at the cell surface**. A. Expression plasmids for full-length WT ST6Gal I (WT), double-mutant ST6Gal I (L37A/K40A) and N-terminal ST6Gal I deletion mutant (ΔN, containing amino acids 43-403) were constructed. WT and L37A/K40A were N-terminally tagged with Flag; ΔN was tagged with HA. Expression of each construct was confirmed by immunoblotting. SW480 cells were transfected with WT, L37A/K40A, or ΔN. Then, ST6Gal I enzymatic activity was measured by ELISA (**B**), and cell surface sialylation was detected by FACS (**C**). Data are presented as means ± SDs of three replicates (*p < 0.05 vs. the corresponding control).

It was previously demonstrated that replacement of ST6Gal I residues Leu37 and Lys40 with alanine significantly decreases BACE1-mediated cleavage of ST6Gal I [34]. Therefore, we prepared a N-terminally Flag-tagged and C-terminally myc-tagged, non-cleavable ST6Gal I (L37A/K40A) mutant and an HA-tagged, N-terminally truncated soluble form of ST6Gal I (ΔN, amino acids 43-403) lacking the protease-specific recognition motif (Figure 2A). We then transiently transfected cells with ST6Gal I WT, L37A/K40A or ΔN constructs, and examined ST6Gal I protein expression by immunoblotting (Figure 2A) and measured ST6Gal I enzymatic activity and sialylation of cell surface proteins 24 hours later. We found that the enzymatic activity of both the non-cleavable L37A/K40A mutant and the ΔN soluble form was similar to that of WT (Figure 2B). In addition, L37A/K40A increased cell surface sialylation to a similar extent as WT. However, in the case of ΔN, which also had enzymatic catalytic activity, cell surface glycoproteins were barely sialylated compared to WT or L37A/K40A (Figure 2C). These results show that the non-cleavable form of ST6Gal I has enzymatic activity and increases α2, 6 sialylation at the cell surface to the same extent as WT. However, it was suggested that the sialylation targets of the soluble form of ST6Gal I were not cell surface proteins. This result may be due to the fact that there isn't much CMP-sialic acid substrate on the outside of the cell.

### Non-cleaved form ST6Gal I shows integrin β1 dependent cell migration

**Figure 3:**
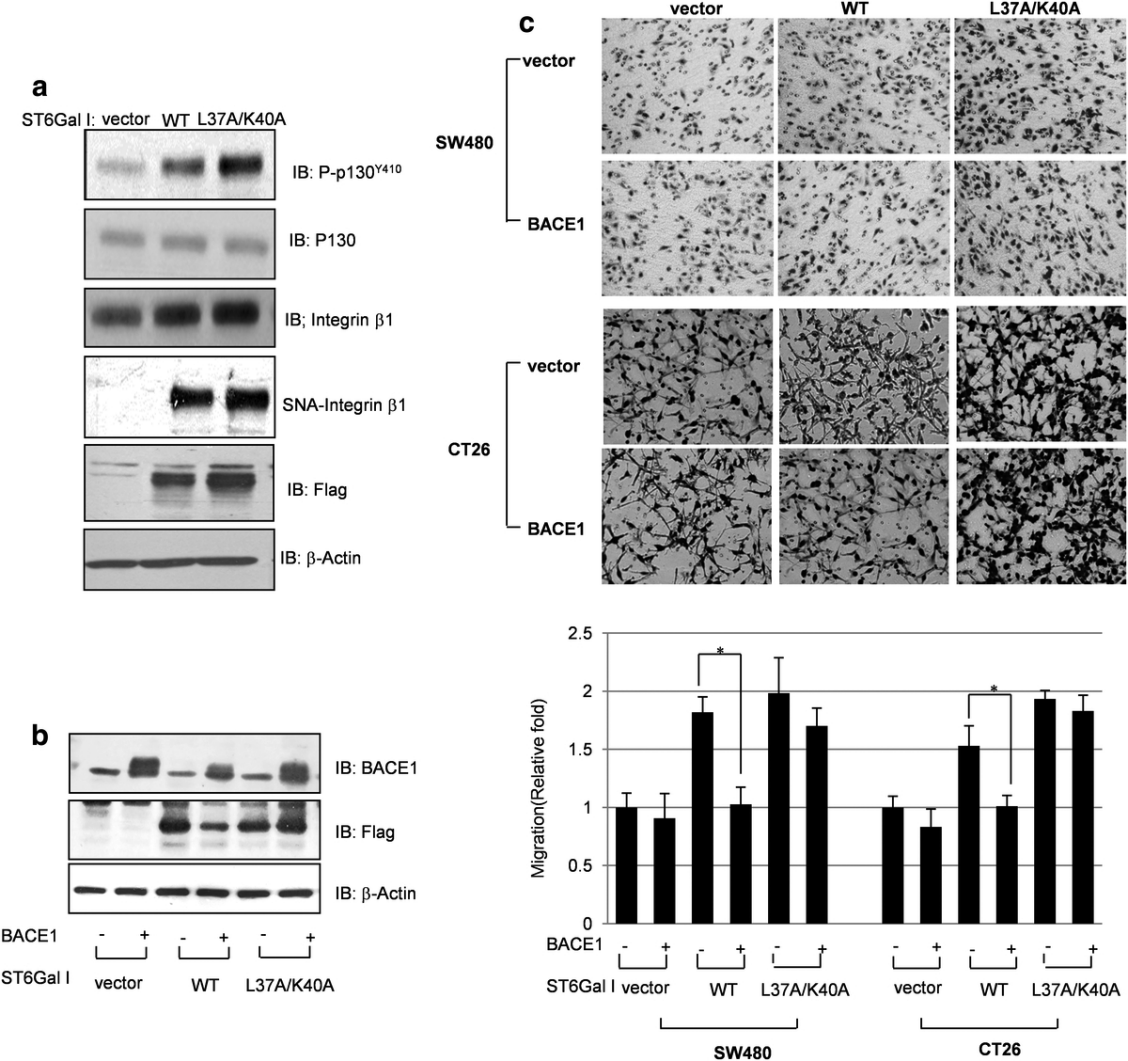
**Sialylation and migration effects of non-cleavable ST6Gal I (L37A/K40A)**. A. SW480 cells were transfected with WT ST6Gal I (WT) or ST6Gal I (L37A/K40A) and cell lysates were analyzed for the phosphorylated form of p130CAS (p-P130CAS^Y410^) and integrin β1 by immunoblotting. Expression of ST6Gal I was assessed using an anti-Flag antibody. Sialylation of integrin β1 was determined by SNA lectin affinity assay. B. SW480 cells were co-transfected with BACE1 and the WT or L37A/K40A form of ST6Gal I. Cell lysates were immunoblotted with antibodies against BACE1 and Flag. C. SW480 cells and CT26 cells were co-transfected with BACE1 and WT or L37A/K40A. Twenty-four hours after transfection, cell migration was determined using Transwell migration assays. *Upper: *images of migrated cells captured by phase-contrast microscopy; *lower: *migration measured by counting cells. Data are presented as means ± SDs of three replicates (*p < 0.05 vs. the corresponding control).

Integrin β1, a known substrate of ST6Gal I [4,38], together with associated adaptor molecules, such as p130CAS, plays a role in promoting the migration of cancer cells [39-41]. We previously reported that integrin β1 sialylation and its associated cancer cell migration-related signaling activity are increased by IR-induced ST6Gal I [22,23,26]. The phenomena described above prompted us to compare the action of WT and non-cleavable L37A/K40A on integrin β1 sialylation, integrin β1-mediated signaling, and cell migration. As shown in Figure 3A, transfection with L37A/K40A increased the Tyr410-phosphorylated (active) form of p130CAS (p-p130CAS^Y410^) and sialylation of integrin β1 to a greater degree than did the WT form. Next, we further examined the effects of BACE1 on ST6Gal I cleavage by co-transfecting SW480 cells with BACE1 and WT or L37A/K40A forms of ST6Gal I. These experiments showed that the Golgi-anchored WT proform was decreased to a greater extent by co-transfection of BACE1 compared with the non-cleavable L37A/K40A form (Figure 3B). We then extended our investigation of the roles of BACE1 in ST6Gal I-mediated migration of cancer cells by co-transfecting SW480 and CT26 cells with BACE1 and WT or L37A/K40A. Cell migration was increased to a much lesser extent by co-expression of BACE1 and WT compared with coexpression of BACE1 and L37A/K40A (Figure 3C). These findings demonstrate that BACE-1 is capable of cleaving the Golgi-membrane anchored ST6Gal I, and indicate that BACE-1-induced cleavage of ST6Gal I results in decreased cell migration.

### The soluble form of ST6Gal I is partially involved in the migration of colon cancer cells

**Figure 4:**
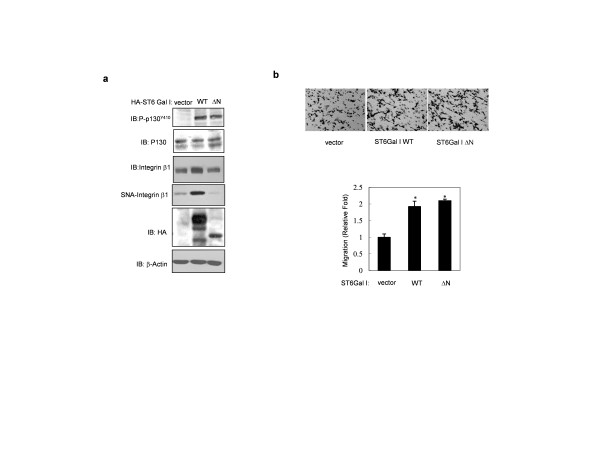
**The soluble form of ST6Gal I increases the migration of colon cancer cells**. A. SW480 cells were transfected with WT ST6Gal I (WT) or ST6Gal I-ΔN (ΔN), and cell lysates were analyzed for the phosphorylated form of p130CAS (p-P130CAS^Y410^) and integrin β1 by immunoblotting. Sialylation of integrin β1 was determined by SNA lectin affinity assay. B. After transfection with the WT or ΔN form of ST6Gal I, cell migration was determined using Transwell migration assays. *Upper: *images of migrated cells captured by phase-contrast microscopy; *lower: *migration measured by counting cells. Data are presented as means ± SDs of three replicates (*p < 0.05 vs. the corresponding control).

Next, we compared the effects of ST6Gal I WT and ΔN on integrin β1 sialylation, integrin β1-mediated signaling, and cell migration. Expression of either WT or ΔN increased the levels of the Tyr410-phosphorylated (active) form of p130CAS (p-p130CAS^Y410^; Figure 4A) and enhanced cell migration (Figure 4B). Although the ΔN soluble form had no significant effects on sialylation of the cell surface glycoprotein integrin β1, it significantly induced p130CAS phosphorylation and migration of SW480 cells (Figure 4A and 4B) and CT26 cells (data not shown), similar to the results of our previous study [23,26]. These findings suggest that the soluble form of ST6Gal I could be involved in p130CAS signaling and migration of colon cancer cells, which, in turn, may be independent of integrin β1 sialylation.

### BACE-1 mediated cleavage and secretion of ST6Gal I are increased by IR

**Figure 5:**
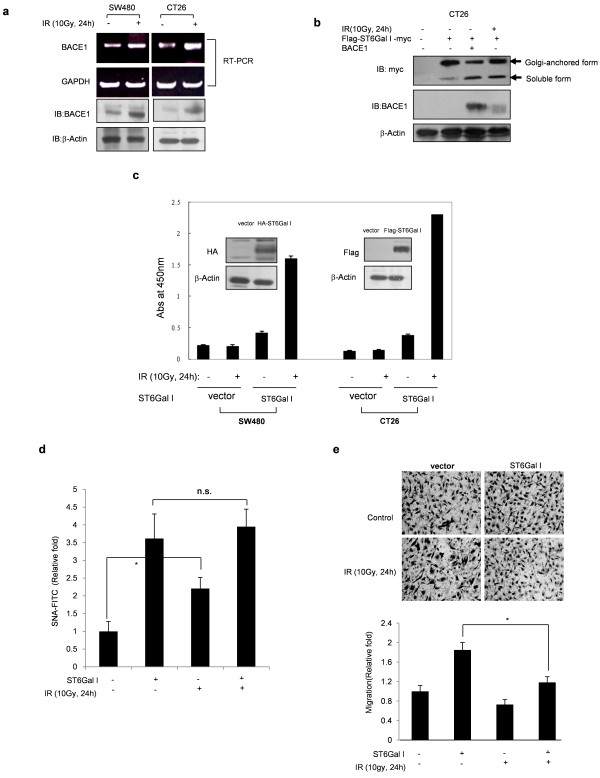
**IR-induced BACE-1 mediates cleavage of ST6Gal I, and inhibits sialylation of cell surface molecules and colon cancer cell migration**. A. Following irradiation of SW480 and CT26 cells, BACE1 expression levels were determined by RT-PCR and immunoblotting. CT26 vector control cells and cells stably expressing ST6Gal I were exposed to 10 Gy of IR. B. The soluble form of ST6Gal I was analyzed after co-transfection with WT ST6Gal I and BACE1, or after exposure of IR (10 Gy, 48 h) to WT ST6Gal I -transfected cells. C. SW480 and CT26 colorectal carcinoma cell lines were stably transfected with ST6Gal I and exposed to 10 Gy of IR. After 24 hours, culture media were harvested and ST6Gal I activity was assayed by ELISA. Data are presented as means ± SDs from three replicates. CT26 vector control cells and cells stably expressing ST6Gal I were exposed to 10 Gy of IR. After 24, α2,6 sialylation of cell surface was analyzed by flow cytometry. Data are presented as means ± SDs of three replicates. *p < 0.05. n.s., not significant. (D). Cell migration was determined using Transwell migration assays (E). *Upper: *images of migrated cells captured by phase-contrast microscopy; *lower: *migration measured by counting cells. Data are presented as means ± SDs of three replicates (*p < 0.05 vs. the corresponding control).

Our previous study indicated that IR induces ST6Gal I cleavage and secretion of a soluble form of ST6Gal I [23]. Because it has been suggested that BACE1 is a protease of ST6Gal I, we tested the effect of IR on BACE1 expression. Importantly, IR (10 Gy) increased the expression of BACE1 at both mRNA and protein levels in SW480 and CT26 colon cancer cell lines (Figure 5A). Next, we examined whether IR induced ST6Gal I cleavage and produced a soluble form of ST6Gal I. As shown in Figure 5B, IR increased cleavage of ST6Gal I and knockdown of BACE1 inhibited this cleavage. In both SW480 and CT26 cell lines, the enzymatic activity of ST6Gal I in culture media was increased compared to that in control cells after exposure of vector control or ST6Gal I-overexpressing cells to IR (Figure 5C). However, even though IR alone slightly increased cell surface protein sialylation which might be induction of ST6Gal I by IR [23,24], Golg-anchored ST6Gal I-mediated increase of cell surface sialylation was not augmented by IR (Figure 5D). We also checked the effect of IR on ST6Gal I-induced cell migration. ST6Gal I enhanced CT26 cell migration, an effect that was partially inhibited by IR. These results indicate that IR acted through induction of BACE1 expression to induce cleavage of ST6Gal I protein to a soluble form that retained sialyltransferase activity. ST6Gal I-induced cell surface sialylation and migration were partially inhibited by IR, an effect that might be due to cleavage of ST6Gal I (Figure 5E).

## Discussion

In the present study, we showed that IR induced cleavage and secretion of the soluble form of ST6Gal I by increasing BACE-1 expression, an effect that may contribute to IR-induced migration of colon cancer cells, which phenomena is independent of integrin β1 sialylation that was usually mediated by Golgi-anchored ST6Gal I.

Our previous study suggested that IR increases the expression of ST6Gal I, which, in turn, is involved in radioresistance and radiation-induced migration via sialylation of integrin β1 [22,23,26]. Another interesting issue raised previously is that IR increases ST6Gal I cleavage and secretion into culture media [23]. While the current data indicate that the Golgi is the main site of sialylation by ST6Gal I in most cells, the fact that soluble forms of ST6Gal I are detected in body fluids and media from cultured cells has raised questions concerning the functions of this extracellular ST6Gal I. It is known that ST6Gal I is not the only transferase that exists in a soluble form; other glycosyltransferases are also detected in the systemic circulation of cancer patients, where they are associated with disease severity and poor prognosis. It has also been suggested that soluble ST6Gal I may be a potential biomarker for the clinical evaluation of colorectal cancer, highlighting the importance of elucidating the function of soluble ST6Gal I, particularly in the radiation-induced migration of cancer cells.

BACE-1, which is a crucial protease in the pathogenesis of Alzheimer's disease, is highly expressed in the brain, but it is also expressed at low levels in peripheral tissues. Information regarding BACE-1 expression and functions outside of the brain is limited. Furthermore, the physiological importance of BACE-1 in cancer progression, metastasis, and responsiveness to radiation remains largely unknown. Evidence that BACE-1 is one of the proteases responsible for the cleavage of ST6Gal I was provided by experiments employing a β-secretase inhibitor or siRNA-mediated knockdown of BACE-1 (Figure 1). The results of these experiments suggest that BACE1 activity involved in cleavage of ST6Gal I from the Golgi membrane and inducing secretion.

Interestingly, inhibition of γ-secretase also increased ST6Gal I levels and cell surface sialylation. Although BACE-1 expression levels could be a major determinant of ST6Gal I cleavage, other regulatory mechanisms might also affect cleavage, secretion, or sorting of ST6Gal I to critical subcellular localizations where ST6Gal I could encounter proteases. Such BACE-1-independent mechanisms might also regulate ST6Gal I function.

In colon cancer cells, IR increased the expression of BACE1, which is involved in IR-mediated cleavage of ST6Gal I (Figure 5). To address the detailed mechanisms linking ST6Gal I to cancer cell migration, we established non-cleavable (L37A/K40A) and soluble (ΔN) variants of ST6Gal I that retained catalytic activity (Figure 2). Cleaved ST6Gal I was secreted into the culture media after exposure to radiation (Figure 5), and the truncated soluble ΔN form retained sialyltransferase activity (Figure 2). We had predicted that the soluble form of ST6Gal I was merely a byproduct of the Golgi-anchored proform of ST6Gal I that was excreted into the extracellular milieu after exposure of cells to radiation. However, ST6Gal I-ΔN also induced cellular migration (Figure 4), suggesting that the soluble form of ST6Gal I could function in tumor cell migration with unknown mechanism. In addition, it was proposed that soluble ST6Gal I would catalyze the sialylation of soluble glycoproteins in the *trans*-Golgi network, or secretory vesicles [35]. These phenomena would be related with sialylation of unknown glycoproteins in intracellular region and subsequently have effect on the process of cell migration. An examination of integrin β1, which is involved in IR-mediated sialylation and tumor migration [23,26], showed that the soluble form of ST6Gal I did not affect the sialylation of integrin β1 (Figure 4). However, cleavage of ST6Gal I by IR inhibited IR-induced migration (Figure 5), indicating that increase of integrin β1-mediated migration by Golgi-anchored ST6Gal I is more potent phenomena than that of soluble form ST6Gal I.

Therefore, it could be suggested that a transfer of sialic acids that is independent of integrin β1 sialylation might be involved in soluble ST6Gal I-mediated migration and activation of p130CAS.

## Conclusion

Collectively, our data suggest that glycoconjugation by the Golgi form of ST6Gal I, possibly in cooperation with soluble ST6Gal I, may be involved in the process of cancer metastasis, especially after radiation therapy.

## Competing interests

The authors declare that they have no competing interests.

## Authors' contributions

ML and JJP performed experiments. YGK and YSL designed, analyzed data, and wrote paper. All authors have read and approved the final manuscript.

## Funding

This work was supported by the Nuclear Research and Development Program through a National Research Foundation of Korea (NRF) grant funded by the Korean government (Ministry of Education, Science and Technology; grant code: M2AMA006), and by a grant from the Korea Healthcare Technology R&D Project, Ministry for Health, Welfare & Family Affairs (grant code: A100627). This work was also supported by the Ewha Global Top5 Grant 2011 of Ewha Womans University.
